# Implantation of Ferumoxides Labeled Human Mesenchymal Stem Cells in Cartilage Defects

**DOI:** 10.3791/1793

**Published:** 2010-04-05

**Authors:** Alexander J. Nedopil, Lydia G. Mandrussow, Heike E. Daldrup-Link

**Affiliations:** Department of Radiology and Biomedical Imaging, Medical Center, University of California San Francisco

## Abstract

The field of tissue engineering integrates the principles of engineering, cell biology and medicine towards the regeneration of specific cells and functional tissue. Matrix associated stem cell implants (MASI) aim to regenerate cartilage defects due to arthritic or traumatic joint injuries. Adult mesenchymal stem cells (MSCs) have the ability to differentiate into cells of the chondrogenic lineage and have shown promising results for cell-based articular cartilage repair technologies. Autologous MSCs can be isolated from a variety of tissues, can be expanded in cell cultures without losing their differentiation potential, and have demonstrated chondrogenic differentiation *in vitro* and *in vivo*^1, 2^.

In order to provide local retention and viability of transplanted MSCs in cartilage defects, a scaffold is needed, which also supports subsequent differentiation and proliferation. The architecture of the scaffold guides tissue formation and permits the extracellular matrix, produced by the stem cells, to expand. Previous investigations have shown that a 2% agarose scaffold may support the development of stable hyaline cartilage and does not induce immune responses^3^.

Long term retention of transplanted stem cells in MASI is critical for cartilage regeneration. Labeling of MSCs with iron oxide nanoparticles allows for long-term *in vivo* tracking with non-invasive MR imaging techniques^4^.

This presentation will demonstrate techniques for labeling MSCs with iron oxide nanoparticles, the generation of cell-agarose constructs and implantation of these constructs into cartilage defects. The labeled constructs can be tracked non-invasively with MR-Imaging.

**Figure:**
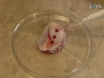


## Protocol

### 1. Labeling of hMSCs with Endorem

Cells are grown to 80% confluence at least 18 hours before labeling.
During this time, labeling media is prepared by adding Endorem to the sample at a dose of 100 ug Fe/ml serum-free media.
After cells are grown to confluence, culture media is aspirated and cells are washed 1x with PBS or serum-free media.
Next, the rinse solution is aspirated and the previously prepared labeling media is added. The cells are then incubated at 37°C, 5% CO_2_ for 4 hours.
After 4 hours incubation, labeling media is aspirated and cells are rinsed with PBS and trypsinized as usual.
Once trypsinized, cells are washed 3x with PBS and centrifuged at 400rcf for 5 minutes.
After this washing step, cells are resuspended, counted, assessed for viability, and used as needed for experimentation.

### 2. Preparing Agarose

After the cells have been labeled it is time to prepare the agarose solution.
On a fine scale, 80mg of agarose powder are weighed and added to 2ml of PBS.
This solution is then gently shaken and autoclaved.
After approximately 40 min, the fluid agarose solution can be taken out of the autoclave. The volume should be measured and adjusted accordingly with preheated PBS.
Then the solution is cooled down to 42°C

### 3. Creating MASI

When the agarose solution has reached the desired temperature, the cells are counted and centrifuged for the last time in a 1.5ml Eppendorf tube.
After centrifugation, the supernatant is discarded and the cells are mixed with the DMEM to reach a concentration of 30 x 10^6^ cells/ml.
Now the cells are mixed with the 42°C warm agarose.
A cold pipette tip may cause agarose to solidify inside the tip, therefore it is advised to preheat the tip by pipetting hot sterile PBS up and down
Now, the same volume of agarose as media is taken up and mixed thoroughly with the cell-media-suspension so that a homogenous suspension is created.
Keep the Eppendorf tube inside a preheated water bath while mixing so that the agarose does not cool down enough to solidify.
When the suspension is homogenous, the cells are implanted in the defect.

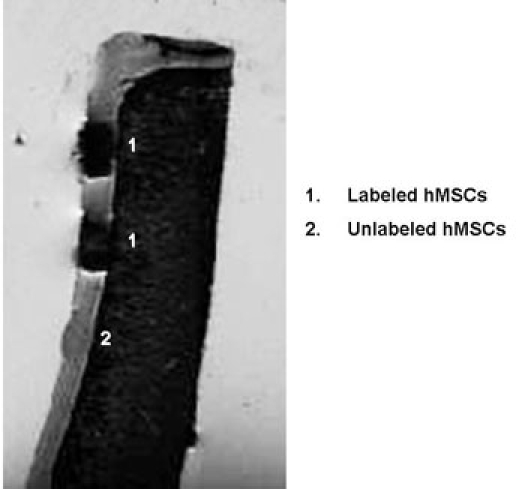
 **Figure 1.** Coronal T2 weighted SE images (TR 4000 ms/ TE 18.27 ms) of a patella specimen with implanted hMSCs in cartilage defects.

## Discussion

The described protocol provides a reproducible way to label MSCs with iron oxide nanoparticles and implant these labeled MSCs into cartilage defects. This technique permits non-invasive depiction of stem cell transplants in cartilage defects with MR imaging, which allows an early detection of MASI failure. A dislocation or efflux of the labeled cells can be diagnosed based on a disappearance of the label from the transplantation site on MR images. An early diagnosis of these events is desirable and would prevent the patient from unnecessary alternative invasive diagnostic procedures. The described non-invasive method for diagnosing MASI outcomes could aid in the successful development of cell based therapies for cartilage regeneration in osteoarthritis. Our protocol is currently applied in preclinical in-vivo studies, would be in principle clinically applicable and could serve as non-invasive outcome measure for the assessment of MASI therapies in clinical practice.
